# Outcomes of Moral Case Deliberation - the development of an evaluation instrument for clinical ethics support (the Euro-MCD)

**DOI:** 10.1186/1472-6939-15-30

**Published:** 2014-04-08

**Authors:** Mia Svantesson, Jan Karlsson, Pierre Boitte, Jan Schildman, Linda Dauwerse, Guy Widdershoven, Reidar Pedersen, Martijn Huisman, Bert Molewijk

**Affiliations:** 1 Centre for Health Care Sciences, Örebro University hospital and School of Health and Medical Sciences, Örebro university, Örebro, Sweden; 2 Karlskoga Hospital, Karlskoga, Sweden; 3 Department of Medicine, Örebro University Hospital, Örebro, Sweden; 4 Centre of Medical Ethics, Department of Ethics, Catholic University of Lille, Lille, France; 5 Institute for Medical Ethics and History of Medicine, NRW Junior Research Group “Medical Ethics at the End of Life: Norm and Empiricism”, Ruhr-University Bochum, Bochum, Germany; 6 Institute for Health and Care Research,VU University Medical Center and Department of Sociology, VU University, Amsterdam, Netherlands; 7 Centre for Medical Ethics, Institute of Health and Society, University of Oslo, Oslo, Norway; 8 Department of Epidemiology & Biostatistics and the EMGO, Dep. Medical Humanities, Vumc & Emgo, Amsterdam, Netherlands

**Keywords:** Clinical ethics, Clinical ethics support, Ethics consultation, Moral case deliberation, Ethics rounds, Health care providers, Questionnaire, Instrument development, Evaluation

## Abstract

**Background:**

Clinical ethics support, in particular Moral Case Deliberation, aims to support health care providers to manage ethically difficult situations. However, there is a lack of evaluation instruments regarding outcomes of clinical ethics support in general and regarding Moral Case Deliberation (MCD) in particular. There also is a lack of clarity and consensuses regarding which MCD outcomes are beneficial. In addition, MCD outcomes might be context-sensitive. Against this background, there is a need for a standardised but flexible outcome evaluation instrument. The aim of this study was to develop a multi-contextual evaluation instrument measuring health care providers’ experiences and perceived importance of outcomes of Moral Case Deliberation.

**Methods:**

A multi-item instrument for assessing outcomes of Moral Case Deliberation (MCD) was constructed through an iterative process, founded on a literature review and modified through a multistep review by ethicists and health care providers. The instrument measures perceived importance of outcomes before and after MCD, as well as experienced outcomes during MCD and in daily work. A purposeful sample of 86 European participants contributed to a Delphi panel and content validity testing. The Delphi panel (n = 13), consisting of ethicists and ethics researchers, participated in three Delphi-rounds. Health care providers (n = 73) participated in the content validity testing through ‘think-aloud’ interviews and a method using Content Validity Index.

**Results:**

The development process resulted in the European Moral Case Deliberation Outcomes Instrument (Euro-MCD), which consists of two sections, one to be completed before a participant’s first MCD and the other after completing multiple MCDs. The instrument contains a few open-ended questions and 26 specific items with a corresponding rating/response scale representing various MCD outcomes. The items were categorised into the following six domains: Enhanced emotional support, Enhanced collaboration, Improved moral reflexivity, Improved moral attitude, Improvement on organizational level and Concrete results.

**Conclusions:**

A tentative instrument has been developed that seems to cover main outcomes of Moral Case Deliberation. The next step will be to test the Euro-MCD in a field study.

## Background

Traditionally, clinical ethics support has been conducted by Clinical Ethics Committees and/or Clinical Ethics Consultation [1,2]. A more recent form of clinical ethics support is Moral Case Deliberation (MCD) [3,4]. MCD is a facilitator-led collective moral inquiry by health care providers into a concrete moral question connected to a real case in their practice [5,6]. Often, MCD implies using a specific conversation method (such as the Dilemma method or the Socratic Dialogue) [6-10]. For the development of the instrument in this study, we use the term MCD as an umbrella term in line with the definition above excluding the specific conversation methods. Related terms that might be used instead of MCD are: Ethics rounds [9-13], Ethical case discussion [14,15] and Ethics reflection groups [16,17]. The MCD facilitator does not function as an ethics expert or consultant [5,6,10]. The expertise of the MCD facilitator supports the joint reasoning process, fostering a sincere, systematic and constructive dialogue and keeping in focus the moral dimension of the case [6].

The main goal of MCD is to support health care providers to manage ethically difficult situations in everyday clinical practice. Empirical investigations are needed to evaluate in which sense this main goal is reached and to understand what outcomes are produced by MCD. Previous literature reveals four gaps that hamper further empirical evaluation.

First, there is *a lack* of MCD evaluation research in general using validated instruments [2,18,19].

Second, existing *quantitative* MCD evaluation research using predetermined evaluation criteria have failed to detect any outcomes [11,20]. The only previous MCD questionnaire published is the Dutch ‘Maastricht Moral Deliberation Evaluation Questionnaire’ [21]. However, this questionnaire was study-specific for a process evaluation of an implementation of MCD in a specific healthcare setting.

Third, the few available qualitative MCD evaluation studies have merely focused on the evaluation of the MCD sessions and little on the perceived outcomes of the MCD on everyday clinical work [7-10,21].

Fourth, there is a lack of clarity and consensus on which outcomes ideally should be reached. This requires a more thorough consideration [2,22]. A lack of consensus does not have to be a problem in itself, since goals of MCD can change in different contexts and over time. At the same time, this complicates selecting outcomes for MCD and developing an instrument. The contextual nature of MCD sessions prevents simple standardisation [22,23]. Therefore, there is a need to study how outcomes differ and in which ways they are context sensitive (for example influenced by workplace, group dynamic behaviour, facilitator, patient cases and culture). This variety regarding outcomes stresses the need for an instrument that takes into account a broad range of different aspects. Results from using such an instrument can further inform and stimulate the discussion of beneficial outcomes of MCD, and thereby stimulate the professional development of MCD (even if a final consensus will not and need not be reached).

In sum, there is both a lack and need for a standardised outcome evaluation instrument for MCD that is developed according to rigid methodological standards and at the same time able to capture outcomes in different contexts. The fact that there is no consensus on MCD outcomes does not diminish the importance of developing a standardized instrument. Instead it makes it even more important to develop an instrument that allows for variety of different MCD outcomes in different contexts. Information on the outcomes of MCD informs about the quality of MCD which, in the end, will be beneficial for the health care providers. Thus, the aim of this study was to develop a multi-contextual evaluation instrument measuring health care providers’ experiences and perceived importance of outcomes of Moral Case Deliberation.

## Methods

A multi-item instrument was developed with the following method of measurement: literature review, collection of items, categorization, a Delphi approach [24] and content validity testing [25-27]. The construct being measured was attitudes and experiences regarding possible outcomes of Moral Case Deliberation, measured through a reflective model [28]. The development process was inductive and did not emanate from predetermined aspects of the construct. An advisory statement including no objection to test the instrument was given by Swedish Regional Ethical Review Board (dnr 2012/34).

### Participants

Overall, 86 participants contributed to the development process of the instrument (Tables 1 and 2). A purposeful sampling strategy was used. First, a Delphi panel of 13 key informants was selected consisting of clinical ethicists and/or ethics researchers from six different countries. Members of the European Clinical Ethics Network (ECEN) [29] were approached, as well as other clinical ethicists, in order to foster variation (Table 1).

**Table 1:** Characteristics of the members of the Delphi expert panel

**Delphi-members**	**Country**	**Profession**	**Ethicist**	**Ethics researcher**	**Female/Male**	ECEN* **member**	**Participation Delphi round**		
							**1**	**2**	**3**
1	France	Philosopher	x	x	M	x	x	x	x
2	France	Lawyer	x	x	M	x	x	x	x
3	Germany	Physician		x	M	x	x	x	x
4	Netherlands	Nurse		x	F		x	x	x
5	Netherlands	Chaplain	x		M		x	x	
6	Netherlands	Health Scientist	x	x	F		x		
7	Norway	Physician	x	x	M	x		x	
8	Sweden	Philosopher	x		M		x		
9	Switzerland	Nurse	x		F		x	x	
10	Switzerland	Nurse	x		F		x	x	x
11	Switzerland	Philosopher	x	x	M		x	x	
12	Switzerland	Physician	x	x	F	x	x	x	
13	Switzerland	Physician	x		F		x	x	

*European Clinical Ethics Network.

**Table 2:** Characteristics of respondents in the content validity testing

**Characteristics respondents**	Step I CVI* **and individual think-aloud**	**Step II CVI and focus group**	**Step III CVI mailed**	**Step IV CVI and individual think-aloud**	**Step V CVI and focus groups**	**Step VI CVI and focus group**	**Step VII CVI and think-aloud**	**Sum mean (range)**
Participants	5	11	16	14	12	5	5	**73**
Female/male	4/1	8/3	11/5	10/4	9/3	3/2	5/0	50/18
Age (years)	53	48	-	54	45	53	45	49 (37–68)
Experience of MCD	3	0	0	0	12	0	0	15
**Profession**								
Nurse	3	4	2	5	2	1	5	22
Head/administrator	1	2	3	3	5	2		16
Ethicists/facilitators	3		4		7	1		15
Physician		2	3	1		1		7
Physiotherapist		3	1	1				5
Chaplain	1		2		2			5
Nurses assistant				3			5	8
Social scientist			2		2			4
Social worker			1	1				2
Other					1			1
**Specialty**								
Medicine		2	2	4	1			9
Surgery	1	2		4				7
Psychiatry		1	3		1			5
Neurology	1	2						3
Home nursing care				4		4	7	15
Paediatrics		2	1					3
Geriatrics/Pall med**			3	1				4
Habilitation					5	1		6
Ethics support service	2				5	1		8
Other	1	2	7	1			3	14
**Country**								
Sweden	5	11		14				30
Netherlands			7		12			19
Great Britain***			9					9
France						5		5
Norway							10	10

*CVI = Content Validity Index [31] **Palliative medicine ***Members of UK Clinical Ethics Network.

Second, 73 participants were approached as representatives of the target population, including healthcare professions collaborating in patient care as well as social scientists, knowledgeable in MCD practice and the everyday English clinical language. They were asked to participate in the process of cognitive and content validity testing. Variation was sought for different professional backgrounds, medical field, countries and level of experience with MCD (Table 2).

### Literature review

An explorative literature review search was conducted by MS to identify possible outcomes of clinical ethics support in general. The review functioned as an inspiration source and was aimed at generating a list of items and identifying outcome domains. A PUBMED search was performed with the MESH term ‘Ethics Consultation’, published 2002 to 2012 and the words ‘Moral Case Deliberation’ or ‘Ethics rounds’ in title or abstracts. Papers were included if they concerned (1) empirical studies of evaluation of Clinical Ethics Consultation, Clinical Ethics Committees or MCD, and (2) conceptual papers about aims of supporting health care providers concerning clinical ethical issues. Reasons for including papers on Clinical Ethics Consultation and Clinical Ethics Committees were that these outcome measures could also be relevant for MCD and also as a response to reported critique by participants of MCDs being too much reflexivity oriented instead of action oriented [10,21].

Articles were excluded if they concerned only ethics consultation for individual physicians, patients and families, research design issues about clinical ethics support and education. No quality assessment of the involved papers was performed since the aim was only to develop a list of possible outcomes.

### Categorization

MS and BM started the categorisation process with the purpose to build a preliminary conceptual model with three measurement levels: items, subdomains and domains. First, MS and BM grouped the various items into domains, independent from each other. This was a bottom up process (rather than top down or theoretically driven). Next, similarities and differences between the two categorizations as a heuristic tool were used until agreement on the measurement levels existed (see Table 3). Similar items were merged or grouped together and then coded into higher abstraction levels of subdomains and domains [30].

**Table 3:** Categorization and item tracking matrix the process of categorisation and reduction of items

**Stages**	**Domains**	**Subdomains**	**Items at each step n**	**Coding agreement**	**Revision of items n**		
					A*	**B**	**C**
Collection of items			96	-	-	-	-
MS and BM categorisation	Reduced moral distress	Emotional support	48	50%	24	36	20
		Better ability to prioritise					
		Received support to handle ethical problems					
	Enhanced ethical climate	Organisation consistent with ethical standards					
		Enhanced collaboration					
		Enhanced communication					
	Concrete resolution in patient cases	Answers or consensus reached in the patient cases					
		Concrete steps taken in the patient cases					
		Improved process for decision making in the patient cases					
	Enhanced moral competence	Improved moral skills					
		Improved moral attitude					
		Improved ethics knowledge					
Delphi round 1:	Enhanced emotional support	(*Subdomains discarded and abstraction level for domains lowered*)	33	25%	6	10	29
	Improved cooperation & communication						
	Better coherence with organizational policy						
	Clear results						
	Improved moral skills						
	Improved moral attitude						
Delphi round 2	Enhanced emotional support	(*Meaning of one domain was changed and three domains were reformulated*)	26	75%	4	6	18
	Enhanced collaboration						
	Improvement on organizational level						
	Concrete results						
	Improved moral reflexivity						
	Improved moral attitude						

*A = merged, B = discarded, C = reformulated.

### Delphi approach

A Delphi approach of three rounds of structured questionnaires to the expert panel was used in order to gain consensus without dominance on the process from specific experts [24]. The purpose was to further test the categorisation of the selected outcome items and the content validity of the items. Consensus was primarily sought for categorisation and relevance of items, but also for the structure of the instrument.

#### *Delphi round 1*

The Delphi panel was asked to independently categorise the various items into subdomains, and subdomains into domains. The participants were also asked to assess the relevance of all items collected from the literature review (both the items included in the categorisation and the items discarded by MS and BM) on a four-point scale; irrelevant to high relevance. In the light of the result, items, subdomains, and domains were merged, discarded or reformulated by MS and BM.

#### *Delphi round 2*

The panel received information on the categorisations and opinions of all members (anonymously) and a questionnaire on the appropriateness of the new version of the categorisation. A four point scale: not (1), somewhat (2), quite (3) and very appropriate (4) was used. In addition respondents were requested to make comments and suggestions of re-categorisation. On the basis of the Delphi categorization rounds, a draft instrument was constructed for content validity testing. Effort was put into reducing ambiguity in item wording and formulating items into everyday language [26].

#### *Delphi round 3*

The panel received nine closed questions regarding adequacy and clarity of the instruction, the questions and the grading of the draft instrument, with possibilities for comments.

### Content validity testing on target population

Cognitive understanding and relevance of the items were tested on 73 persons from the target population (see Table 2). The testing was conducted through seven steps with different groups of participants at each step, using ratings of content validity index (CVI) and audio recorded ‘think-aloud’ interviews. Revisions were made between each step.

#### *Content Validity Index testing*

The inter-rater agreement of clarity of the items was measured by CVI using the four-point scale according to Yaghmaie: Not clear (1), Item needs some revision (2), Clear but needs minor revision (3) and Very clear (4). The relevance was measured on a four-point scale from irrelevant to high relevance [31]. The rater also had the opportunity to add comments. The questions on simplicity and ambiguity were discarded, after a ‘think-aloud’ pilot test that showed that the respondents gave identical answers on these two aspects, compared to the answers on clarity. Both the appropriateness of categorisation and the CVI was computed according to Polit et al. as universal agreement (S-CVI). An item-CVI (I-CVI) lower than .78 for was considered as a reason for revision [27].

#### *‘Think-aloud’ interviews*

The cognitive method ‘think-aloud’ [25,26] was used while respondents simultaneously filled in the CVI- Through. individual- and focus group interviews [30], the understanding was tested for consistence and in accordance with the way the items were intended to be perceived [25,26]. The interviewers read the items aloud and encouraged the informants to express their meaning with the question: ‘Can you rephrase this item in your own words?’ Furthermore, the participants were encouraged to express opinions on the relevance of the items.

## Results

The European Moral Case Deliberation Outcome Instrument (Euro-MCD) was developed in accordance with the following steps.

### Literature review

The literature search yielded 1378 articles. After reviewing titles according to the inclusion criteria, the number was reduced to 352 papers. After reading the abstract or, in case of insufficient information, the full text, 87 papers which fulfilled the relevant criteria were included for further analysis. The major content of these papers were conceptual and empirical studies on Clinical Ethics Consultation and 15 addressed MCD (umbrella term). At the end of the development process, the final items referred to 21 papers, where nine addressed MCD (Table 4).

**Table 4:** Final model of categorisation

**Domains**	**Items**	**References**
Enhanced emotional support*	1. Enhances possibility to share difficult emotions and thoughts with co-workers	[5,8,10]
	2. Strengthens my self-confidence when managing ethically difficult situations	[10]
	3. Enables me to better manage the stress caused by ethically difficult situations	[6,8,23,32]
	4. Increases awareness of my own emotions regarding ethically difficult situations	[5,14]
	5. I feel more secure to express doubts or uncertainty regarding ethically difficult situations	[6,8,33,34]
Enhanced collaboration	6. Greater opportunity for everyone to have their say	[8,10]
	7. Better mutual understanding of each other’s reasoning and acting	[8,10,21]
	8. Enhances mutual respect amongst co-workers	[2,14,21]
	9. I and my co-workers manage disagreements more constructively	[1,35]
	10. More open communication among co-workers	[2,8,10,21]
Improved moral reflexivity	11. Develops my skills to analyse ethically difficult situations	[1,6,15,35,36]
	12. Increases my awareness of the complexity of ethically difficult situations	[9,21]
	13. Develops my ability to identify the core ethical question in the difficult situations	[10,15,21,34,37]
	14. I see the ethically difficult situations from different perspectives	[8-10,14,21,38]
	15. Enhances my understanding of ethical theories (ethical principles, values and norms)	[15,34]
Improved moral attitude	16. I become more aware of my preconceived notions	[6,10,12]
	17. I gain more clarity about my own responsibility in the ethically difficult situations	[9,10,12]
	18. I listen more seriously to others’ opinions	[14,21]
	19. Gives me more courage to express my ethical standpoint	[21]
	20. I understand better what it means to be a good professional	[21]
Impact on organizational level	21. I and my co-workers become more aware of recurring ethically difficult situations	[33,35]
	22. Contributes to the development of practice/policies in the workplace	[21]
	23. I and my co-workers examine more critically the existing practice/policies in the workplace/organization	(new)
Concrete results	24. Find more courses of actions in order to manage the ethically difficult situation	[34,35,39,40]
	25. Consensus is gained amongst co-workers in how to manage the ethically difficult situations	[9,15,40]
	26. Enables me and my co-workers to decide on concrete actions in order to manage the ethically difficult situations	[2,15,36,37]

*References [11-13].

### Collection of items

A total of 96 preliminary items were collected from the contents of 53 of the 87 papers. In the first reduction step, items were reduced by MS and BM to 48 (Table 3) based on six criteria: similar items, not relevant enough for MCD, too academic, too vague, too unspecific to be categorised to only one domain, too abstract or difficult to measure (Table 5).

**Table 5:** Reasons for discarding items

**Reasons for discarding items**	**Examples of discarded items**
Not relevant enough for MCD	Reduce feelings of bad conscience
	Diminish fear of legal liability
Too unspecific; not possible to categorise to only one domain	Strengthen confidence to see a way out
	Improve ability to prioritise between clinical tasks in an ethical way
Too vague	Improve quality of care in patient cases
	Become aware of the tension between my personal values and actual behaviour
Too abstract	Enhance moral sensitivity
	Enhance knowledge of virtue ethics (such as courage, tolerance, compassion, honesty, humility)
Too academic	Know how to differentiate between ethical issues and other issues (such as psychological or legal)
	Enhance knowledge how to apply the four ethical principles: respect for autonomy, non-maleficence, beneficence and justice

### First categorization

The resulting 48 items were sorted into 12 subdomains, which were attributed into four overarching thematic domains: ‘Enhanced ethical climate’, ‘Enhanced moral competence’, ‘Reduced moral distress’ and ‘Concrete resolution’. For agreement, item reduction and domain revision see the tracking matrix in Table 3.

### Delphi approach

#### *Delphi round 1*

The first round resulted in low agreement on the categorisation of the 48 items. Several of the Delphi-members had categorised items into several subdomains. For example the item ‘Moral consensus for care achieved in the patient cases’ was sorted into the following subdomains, depending on the Delphi-member: ‘Concrete steps taken in the patient cases’, ‘Organisation consistent with ethical standards’ and ‘Enhanced collaboration’. This led to the decision to discard these initial domains and create new ones. In order to gain clarity, all subdomains were discarded and the main domains were made less abstract. The second step of item reduction resulted in 33 items (Table 3).

Regarding the question of relevance of the items, there was also great variability of opinions of the 48 items in the categorisation and of the 54 items discarded by MS and BM from literature review. There was only agreement of high relevance for 10 items and agreement of low relevance for 37 items. No differences of judgments were detected between professions or countries.

#### *Delphi round 2*

In the second round, the appropriateness of the revised set of 33 items and the new categorisation was tested. The agreement between Delphi-members proved to be better (Table 3). The most important change after the second round was that the name of the domain ‘Improved moral skills’ was changed into ‘Improved moral reflexivity’, including moving one item into this domain and discarding two others from it. The fourth and final step of item reduction resulted in 26 items (Table 4).

#### *Delphi round 3*

The third round resulted in 100% agreement regarding the structure of the instrument; adequacy and clarity of the content of instruction, questions and also the response alternatives. After content validity testing, an additional question in both the Euro-MCD I and II was added: ‘Please list five of the most important outcomes’. This was done since the majority of items were viewed as “very important”.

### Content validity testing

#### *The Content Validity Index testing*

The CVI, tested mostly on the target population (Table 2) through seven steps, showed that clarity increased from 0.71 to 0.96 and relevance from 0.70 to 0.95, although the change in relevance was less in France ( 0.77) (Table 6). The think-aloud interviews mainly provided input on clarity for everyday language, highlighted ambiguities and questioning of relevance of certain items. The testing led to 35 major reformulations and 65 minor reformulations and the discarding of two items (Table 6). No differences of judgments were detected between professions or specialties.

**Table 6:** **The content validity** (**CVI**) **tracking matrix**

Content Validity Index CVI* **(n items CVI** **> .78/total n items)**			**Revision**	**Major result of think-aloud interviews and mailed comments influencing the revision process**
Steps	Clarity	Relevance	A /B /C**	
I: CVI and individual think-aloud	.71 (13/26)	.70 (14/26)	7/12/0	Input of reformulations into more concrete language with reductions of double meanings for some of the items.
II: CVI and focus group interview	.73 (15/26)	.70 (13/26)	5/17/0	Input of reformulations of language, grammar, differentiation of group- and individual outcomes and adding “ethics” to some items. Foremost, items belonging to the domains ‘Improvement on organizational level’ and ‘Concrete results’ were questioned.
III: Mailed CVI	.88 (23/26)	.77 (14/26)	11/9/2	Input of sharper English everyday formulations from the British respondents. The Dutch respondents foremost pointed out overlapping items, but different ones pointed out. The items “Reach answers what the ethical problems are” and “Learn to formulate questions about ethical issues” were discarded, due to low clarity and relevance. Two items were brought back: “Enhances my understanding of ethical theories (ethical principles, values and norms)” and “I understand better what it means to be a good professional”.
IV: CVI and individual think-alouds	.94 (21/26)	.85 (23/26)	0/2/0	In the Swedish translation, there were still ambiguous interpretations of the following items: 15, 20, 22, 23, 25 (Table 4). The critique centred on that policy work does not reach staff working on the floor and also on the interpretation what a “good professional” is. Reformulations followed of number 22 and 23, also in the English version.
V: CVI and focus groups	.91 (24/26)	.87 (20/26)	5/12/0	In the Dutch translation, the participants of the focus groups were positive about the clarity and the relevance of the items, although some thought some were still rather abstract. Furthermore, some thought that some items did not fit within their view on MCD. Some problems of clarity were interpreted to be related to the translation into Dutch. Ambiguous interpretations of item 6 and 15 (Table 4).
VI CVI and focus group	.96	.77	3/6/0	In the French focus group, participants were able to see both the English and French version. Three types of reformulation input: grammatical precision, formal clarity and substantial clarification of terms where relevance was questioned. For example, the item 22 (Table 4) became in French “contribute to the evolution of individual and collective practices”, recognizing the importance of the political dimension included in the care activity. Somewhat ambiguous interpretation of 6, 13, 15, 20 and 23.
VII CVI and individual think-alouds	.95	.95	4/7/0	The Norwegians questioned foremost the clarity of the items 20 and 23, especially the meaning of being a good professional. Revison was conducted after answers from five respondents and after next five, the CVI was improved.

*S-CVI = the proportion of each item that achieved rating 3 or 4 divided with the number of respondents and then the average of the item-CVIs (I-CVI) [27].

**A = Major reformulation B = Minor reformulation C = Replaced.

#### *The ‘think-aloud’ interviews and comments*

The interviews and comments led to reformulations of the items, since vague items with ambiguous meanings were pointed out (Table 6). When the informants were uncertain of the meaning of an item, they often gave suggestions for reformulations. See summaries of the interviews in Table 6.

#### *Translation and CVI*

The original language of the instrument is English. Euro-MCD I and II were literally translated into Swedish, Dutch, French and Norwegian by two independent translators for each of the languages. CVI and ‘think-aloud’ interviews were then tested in each country (Table 6). Next, the versions were back-translated into English by other translators [41]. Finally, all the back-translations were compared regarding literal translation and cultural adaption and then harmonized against the English source [41,42]. The back-translations showed high agreement, except for the two items in the domain Impact on organizational level. The the terms ‘practice/policies’ were culturally translated in France and Norway, but literally in Sweden and Netherlands.

### The final instrument

The Euro-MCD consists of two sections. Section I is to be used before MCD and consists of an open question on important outcomes in general, followed by one closed question regarding the importance of the 26 pre-formulated outcomes (the items). Section II is to be used after multiple MCDs. It starts with open questions on experiences of outcomes, followed by a series of closed questions on both the importance and the experience of outcomes (Table 7). A four-point response scale is used for measuring the importance and the experience of outcomes: none, somewhat, quite high and high.

**Table 7:** **Open and closed questions in the two sections of the Euro**-**MCD instrument**

**Euro-MCD I (before the first MCD)**	
1. Imagine participating in Moral Case Deliberations. Please formulate in your own words 3 to 5 outcomes that you consider important to reach in order to support you and your co-workers in managing ethically difficult situations* in everyday clinical practice.	**Open question**
*Regarding the 26 items*:	**Closed**
2. How important is each of the outcomes to you?	‘Very’
	‘Quite’
	‘Somewhat’
	‘Not’
	‘Cannot take stand’
3. Please list 5 of above outcomes that you consider as most important.	**Open question**
**Euro**-**MCD II (after multiple MCDs)**	
1. Please write down in your own words outcomes of the MCD that you have experienced during the MCD meeting(s)	**Open questions**
2. Please write down outcomes of the MCD that you have experienced afterwards, in the everyday clinical practice at your workplace	
*Regarding the 26 items*:	**Closed**
3. Have you experienced the outcome during the MCD meeting(s)?	‘In high degree’
4. Have you experienced it after the MCD, in the everyday clinical practice?	‘In quite high degree’
5. How important is the outcome to you? (Very, quite, somewhat, not, cannot take stand)	‘In some’
	‘Not at all’
	‘Cannot take stand’
6. Please list 5 of above outcomes that you consider as most important (irrespective whether you have experienced them or not).	**Open questions**
7. What should be improved during the MCD meetings?	

*ethically difficult situation was defined as “situations in which you experience unease or uncertainty of what is right or good to do or are in disagreement about what should be done”.

The measurement model comprises 26 items and six domains: Enhanced emotional support, Enhanced collaboration, Improved moral reflexivity, Improved moral attitude, Impact on organizational level and Concrete results (Table 4). Some domains are complex and thus need more items, such as ‘Enhanced emotional support’, containing five items. More simple and concrete domains, such as ‘Concrete results’, contain three items.

## Discussion

Through a systematic process, an instrument was developed measuring a wide range of possible outcomes associated with Moral Case Deliberation in different countries: The Euro-MCD.

The six domains in the final version - Enhanced emotional support, Enhanced collaboration, Improved moral reflexivity, Improved moral attitude, Impact on organizational level and Concrete results - seem to cover main outcomes of MCD. The domains in the initial development process contained only aspects that are directly related to ethics (Table 4). In the final version, the domains: Enhanced emotional support and Enhanced collaboration demonstrate that reflecting (in MCD) on the morally good thing to do also involves dealing with emotions and collaboration. Furthermore, outcomes need not always be of a strictly ethical nature in order to be considered important and relevant by MCD users. This is also described in the literature on MCD [43,44].

Domains similar to those in the Euro-MCD are to be found in other validated instruments, developed outside the area of clinical ethics support. Examples are instruments concerning moral distress [45-47], nurse-physician collaboration [48], ethical climate [46] and team reflexivity [49]. However, all these instruments measure only one specific domain. With the Euro-MCD instrument we want to combine several domains and relate them specifically to MCD. Furthermore, we first want to know *if* there is a systematic pattern of MCD outcomes within the Euro-MCD, and if so, *which* MCD outcomes are most relevant. After testing the Euro-MCD in various countries, and further improving its validity, existing validated instruments mentioned above might be used in combination with the Euro MCD in order to evaluate to what degree the other instruments and the Euro MCD measure the same construct.

A crucial aspect of the instrument is that it measures both what the health care providers’ perceive as important and what they actually experience. It assumes that changes in daily work after MCD sessions, reported by respondents, are *related to* the participation in the MCD sessions. Again, this is the subjective experience of the respondents, and not in any way objectively observed. We think, however, that this in itself is valuable and informative, because the outcomes from the Euro-MCD are experiential phenomena.

A strong point of the instrument is the multinational and multi contextual development process [42] and thus can be applied in a wide variety of contexts. It might even be used in a wider sense, such as evaluating classic ethics consultation. Furthermore, the instrument can be used to measure changes over time through analysing differences in perceived importance before and after multiple MCDs.

We think the instrument can be useful, not only for those who facilitate MCD, but also for those who implement it (e.g. managers, board of directors). The information about the MCD outcomes could be an indicator of the effectiveness and indirectly also of the quality of the MCD at the local and institutional level. We think sharing the results of the Euro-MCD with participants is important for testing the validity and the meaning of these results but also, and especially, to empower MCD participants to use the MCD for their specific interests (i.e. to make them responsible and active owners of MCD).

Results of the Euro-MCD might also be used to develop new ways of facilitation, since low ratings on questions on experiencing certain outcomes during the MCD sessions can stimulate the facilitator to change focus when facilitating future MCD’s. When for example, the rating on ‘better mutual understanding of each other’s reasoning and acting’ receives a low rating, the facilitator could focus more on whether participants understand each other.

### Methodological considerations

The development of the Euro-MCD was a complex, iterative process with continuous revision of items and domains. Refering to Brod’s parable of the GPS system [30], the initial course was plotted by the literature review, but the Delphi-panel and especially the content validity testing corrected the wrong turns in order to make the final destination. Five recommended steps out of six steps in the development of a measurement instrument, as described by de Vet et al. [28] was followed. The last step, field-testing will be conducted with the current version of the Euro-MCD. The study’s strength lies in the comprehensive methodology in developing the Euro-MCD and the use of standard statistical assessment at each stage. However, it might be considered as a weakness that this instrument does not measure objective outcomes (e.g. related to sick leave, turnover rates of employees, and patient related outcomes). Objective outcomes are important, but seem to be more difficult to capture and require another kind of research instrument and research design.

We selected a Delphi panel of members with high levels of expertise [27], since the members were MCD facilitators and/or ethics researchers from different countries and with different professional backgrounds. There was also a wide variation in respondents for the content validity process. However, a potential bias might has been caused by the fact that most respondents were Swedish, lacked experience of MCD and were middle-aged. When testing the instrument before and after multiple MCDs in the coming field study, we will also capture younger respondents with experience of MCD from four European countries.

The process of categorization was complicated by the diversity of opinions of the Delphi expert panel. As a consequence, this process did not give sufficient guidance for the categorization and item reduction. On the one hand, this is an interesting result in itself: experts on clinical ethics support vary on what kind of outcomes they consider appropriate for MCD. On the other hand, the low agreement between the Delphi-expert panel in the first round could be a result from the open and imprecise nature of the assignment given to them. In hindsight, we should have informed the Delphi expert panel more clearly that the domains were seen as *reflections* of the items (reflective model), not as *predictions* of the items (formative model) [28]. In Delphi round 2, the agreement of the categorization made by MS and BM was higher and the comments were much more distinct and helpful. It might be concluded that the first round was too open, and the second one too narrow.

The development of the Euro-MCD is not finished. In the coming field-study, health care providers have the opportunity to add items through the open questions in the instrument. This provides the opportunity for considering possible neglected but important outcomes in the next version. The open questions also fit with our contextual and pragmatic viewpoint that the specific context should have a say in which specific goals and outcomes of MCD are important. However, we are aware of that the number of items is too extensive and our goal is to decrease them after the field-study. In this phase it could be hazardous to limit them too much.

The content validity process was successful. This indicates that we have captured and formulated clear and relevant items, even though it was tested in only fiveWestern European countries. According to Brod et al., combining focus-groups and individual interviews support the content validity [30]. The focus group interview with healthcare professions working both clinically and as researchers was especially successful. The open and vivid communication enabled participants to equally express their opinions. Thus, a rich variation of relevance judgments and substantial suggestions for reformulations were generated. The discussion of the relevance of the item focusing on reaching consensus was especially salient. There were both strong opinions against, in that the consensus reached may be unethical, and strong opinions in favor, in that consensus are needed for a concrete problem in a particular patient situation.

We reached the recommended goal of a mean Content Validity Index over .90, as recommended by Polit et al. [27]. This means that the validity of the current items can be considered as high. However, questions can be raised concerning discriminative validity, since some items overlap. However, different respondents perceived different items as similar. After the results of the field study, items that overlap will be merged or deleted.

## Conclusions

A tentative measurement model was developed that seems to cover main outcomes of Moral Case Deliberation. Both the process and the result of the content validity procedures seem high. The Euro-MCD is unique since it is flexible enough to be tailored to the specific needs of local MCD groups, but general enough to capture relevant MCD outcomes across multiple contexts.

However, a broader field study is needed to further develop and validate the Euro-MCD, collecting data from participants who have experienced multiple MCDs. Such a study will be conducted in France, Netherlands, Norway and Sweden. Psychometric testing will be used to evaluate the construct validity of the measurement model. This testing might lead to further reduction of items, but also to addition of new ones. Further aims are to find out whether systematic patterns of experienced outcomes and perceived importance can be found, or whether the results will be context sensitive.

In order to further develop and validate the instrument, those who want to use the Euro-MCD, please contact the first or last author.

## Competing interests

The authors declare that they have no competing interests.

## Authors’ contributions

MS and BM initiated and coordinated the study. MS carried out the literature review, categorization, Delphi approach and content validity testing and drafted the manuscript. BM participated in the design of study, the categorization, the data-collection, and drafting the manuscript. All authors read and approved the final manuscript. JK participated substantially in the design of the study and writing the manuscript. PB participated in the data-collection, the Delphi rounds, made substantial contributions to the categorisation and also gave comments on the writing of the manuscript. JS and LD participated in all the Delphi rounds and contributed with substantial comments in the writing of the manuscript. GW and MH made contributions to the design of the study and made substantial contributions to the manuscript. RP participated in one Delphi round and made substantial contributions to the writing of the manuscript.

## Pre-publication history

The pre-publication history for this paper can be accessed here:

http://www.biomedcentral.com/1472-6939/15/30/prepub
